# Current Insights on Dyslipidaemia Management for Prevention of Atherosclerotic Cardiovascular Disease: A Malaysian Perspective

**DOI:** 10.21315/mjms2023.30.1.6

**Published:** 2023-02-28

**Authors:** Wan Azman Wan Ahmad, Azhari Rosman, Sunita Bavanandan, Mafauzy Mohamed, Muhamad Ali Sk Abdul Kader, Tamil Selvan Muthusamy, Kai Huat Lam, Sazzli Shahlan Kasim, Fan Kee Hoo, Mayuresh Fegade, Zhi Ling Looi, Abdul Rashid Abdul Rahman

**Affiliations:** 1 Department of Medicine, Universiti Malaya Medical Centre, Kuala Lumpur, Malaysia; 2 Cardiology, National Heart Institute, Kuala Lumpur, Malaysia; 3 Department of Nephrology, Kuala Lumpur Hospital, Kuala Lumpur, Malaysia; 4 Department of Endocrinology, Hospital Universiti Sains Malaysia, Kelantan, Malaysia; 5 Department of Cardiology, Serdang Hospital, Selangor, Malaysia; 6 Cardiology, Cardiac Vascular Sentral, Kuala Lumpur, Malaysia; 7 Cardiology, Assunta Hospital, Selangor, Malaysia; 8 Faculty of Medicine, Cardiac Vascular and Lung Research Institute, Universiti Teknologi MARA, Selangor, Malaysia; 9 Department of Medicine, Faculty of Medicine, University Putra Malaysia, Selangor, Malaysia; 10 Novartis Corporation (Malaysia) Sdn. Bhd., Selangor, Malaysia; 11 Department of Medicine, An-Nur Specialist Hospital, Selangor, Malaysia

**Keywords:** dyslipidaemia, atherosclerotic cardiovascular disease, low-density lipoprotein cholesterol, statin, lipid-lowering therapy

## Abstract

Dyslipidaemia is highly prevalent in the Malaysian population and is one of the main risk factors for atherosclerotic cardiovascular disease (ASCVD). Low-density lipoprotein cholesterol (LDL-C) is recognised as the primary target of lipid-lowering therapy to reduce the disease burden of ASCVD. Framingham General CV Risk Score has been validated in the Malaysian population for CV risk assessment. The Clinical Practice Guidelines (CPG) on the management of dyslipidaemia were last updated in 2017. Since its publication, several newer randomised clinical trials have been conducted with their results published in research articles and compared in meta-analysis. This underscores a need to update the previous guidelines to ensure good quality care and treatment for the patients. This review summarises the benefits of achieving LDL-C levels lower than the currently recommended target of < 1.8mmol/L without any safety concerns. In most high and very high-risk individuals, statins are the first line of therapy for dyslipidaemia management. However, certain high-risk individuals are not able to achieve the LDL-C goal as recommended in the guideline even with high-intensity statin therapy. In such individuals, lower LDL-C levels can be achieved by combining the statins with non-statin agents such as ezetimibe and PCSK9 inhibitors. Emerging non-statin lipid-lowering therapies and challenges in dyslipidaemia management are discussed in this article. The review also summarises the recent updates on local and international guidelines for dyslipidaemia management.

## Introduction

Cardiovascular diseases (CVDs) are the most common cause of mortality around the world (1–3). Major cardiovascular risk factors include dyslipidaemia, hypertension, smoking, diabetes, obesity, and physical inactivity. The global prevalence of elevated total cholesterol was estimated to be 11.9% (4). Elevated levels of total cholesterol, triglyceride, low-density lipoprotein cholesterol (LDL-C) and low levels of high-density lipoprotein cholesterol were highly prevalent in the Malaysian population (5). Current data on the safety of very low LDL-C supports the hypothesis that ‘lower is better’ (6).

In Malaysia, CVDs have been reported as the leading cause of death among non-communicable diseases (NCD) (7, 8). The results of the National Health and Morbidity Survey (NHMS) published in 2019 found one in four people mostly in the age group of 40 years old–59 years old to be unaware of their hypercholesterolemia status (9). The Malaysian National Cardiovascular Disease Database (NCVD) registry showed 54.8% of patients with percutaneous coronary intervention (PCI) and 38.6% of patients with the acute coronary syndrome (ACS) had hypercholesterolemia (10, 11).

Elevated LDL-C levels in the blood are responsible for accumulation and retention of LDL particles in the endothelial wall leading to accelerated atherosclerotic plaque progression and rupture. The thrombus associated with plaque rupture obstructs the coronary blood vessel leading to adverse cardiovascular (CV) events (12). For this reason, plasma LDL-C levels have been linked to the development of atherosclerotic cardiovascular disease (ASCVD) (13, 14). Several clinical trials have shown that lowering LDL-C level reduces CV events (15–17). Being a positive risk factor for ASCVD, most guidelines identify LDL-C as a target of treatment in dyslipidaemia and CVD patients (18). The Malaysian Clinical Practice Guidelines (CPG) on the Management of Dyslipidaemia and CPG on Primary and Secondary Prevention of CVD were last updated in 2017.

Since 2017 many newer clinical trials, meta-analyses and review articles have been published on this topic, thus there is a pressing need for an update. The objective of this review is to provide current insight on the management of dyslipidaemia to ensure optimal care and outcome for our patients.

### Literature Search Strategy

This narrative review is based on literature search carried out through PubMed and Google Scholar. English language publications available until 20 July 2021 were searched by combining the following search terms using Boolean operators ‘AND/OR’: ‘Malaysia’, ‘South East Asia’, ‘Cholesterol’, ‘LDL’, ‘Total Cholesterol’, ‘Dyslipidaemia’, ‘Hypercholesterolemia’, ‘Cardiovascular Disease’, ‘ASCVD’, ‘Diabetes’, ‘Chronic Kidney Disease’, ‘Atherosclerosis’, ‘Stroke’, ‘Cardiovascular Events’, ‘Major Adverse Cardiovascular Events’, ‘Risk Factors’, ‘Framingham Risk Score’, ‘Atherosclerotic Plaque’, ‘PCSK9 Inhibitor’, ‘Safety’, ‘Efficacy’, ‘Adverse Events’, ‘Acute Coronary Syndrome’, ‘Lipid Lowering Therapy’, ‘Combination Therapy’, ‘Statin’, ‘Evolocumab’, ‘Alirocumab’, ‘Ezetimibe’, ‘Ischaemic Attack’ or ‘Cumulative LDL-C Exposure’.

### Cardiovascular Risk Assessment

The possibility of an individual experiencing a CV event, whether fatal or non-fatal, over a given period is referred to as CV risk (19). The basis of current approaches to primary CVD prevention is the estimation of the absolute risk of acquiring an incident of CVD event. The available literature describes several risk equations and risk models to assess CV risk. All CV risk assessment models face limitations and challenges during extrapolation to different populations. Preferably, the adopted CV risk model should be remodeled based on data pertaining to the local population to define the risk categories (20). The Framingham Risk Score-Cardiovascular Disease (FRS-CVD) for 10-year CV risk calculation for CVD risk stratification has been found to be more accurate than the Framingham Risk Score-Coronary heart disease (FRS-CHD), QRESEARCH cardiovascular risk algorithm score (QRISK2), Joint British Society risk calculator 3 (JBS3), ACC/AHA atherosclerotic cardiovascular disease (ASCVD) risk score and WHO/ISH risk charts (21). The FRS-General CVD risk score tool has been developed from a treatment-naive population or those subjected to minimum treatment. In addition, FRS-CVD is a straightforward and simple to use tool (20). The FRS and Systematic Coronary Risk Evaluation (SCORE) were found to be efficient in identifying patients at high risk for CVD in a local study from Malaysia (22). The FRS-General CVD risk score tool has good calibration and discriminates moderately well for the Malaysian population (23). Hence FRS-General CVD risk score tool is highly recommended for assessing CV risk in the Malaysian population (20).

### Cardiovascular Risk Stratification

Based on CV risk stratification, patients are categorised into low risk, moderate risk, high risk and very high-risk groups by the 2017 Malaysian CPG on the Management of Dyslipidaemia and the 2019 ESC/EAS Guideline on the Management of Dyslipidaemias (20, 24). Meanwhile, the 2018 ACC/AHA Guideline on the Management of Blood Cholesterol focuses on cholesterol management in very high-risk ASCVD patients (25) (Table 1).

### Malaysian Guidelines on Dyslipidaemia Management

Presently two major CPGs are available in Malaysia—CPG on the Management of Dyslipidaemia (20) and CPG on Primary and Secondary Prevention of CVD (26). According to the 2017 Malaysian CPG on the Management of Dyslipidaemia, target LDL-C levels are based on the patient’s risk profile for CVD. For patients with very high CV risk, the target LDL-C should be < 1.8 mmol/L or > 50% reduction from baseline. For high CV risk patients, LDL-C of ≤ 2.6 mmol/L or > 50% reduction from baseline is recommended. Target LDL-C of < 3.0 mmol/L is recommended for patients with intermediate/moderate and low CV risk. Similar LDL-C goals were recommended by the 2017 CPG on Primary and Secondary Prevention of CVD (20, 26).

### Evolution of Dyslipidaemia Management

#### Overview of Dyslipidaemia Management Algorithm

**Figure 1:**
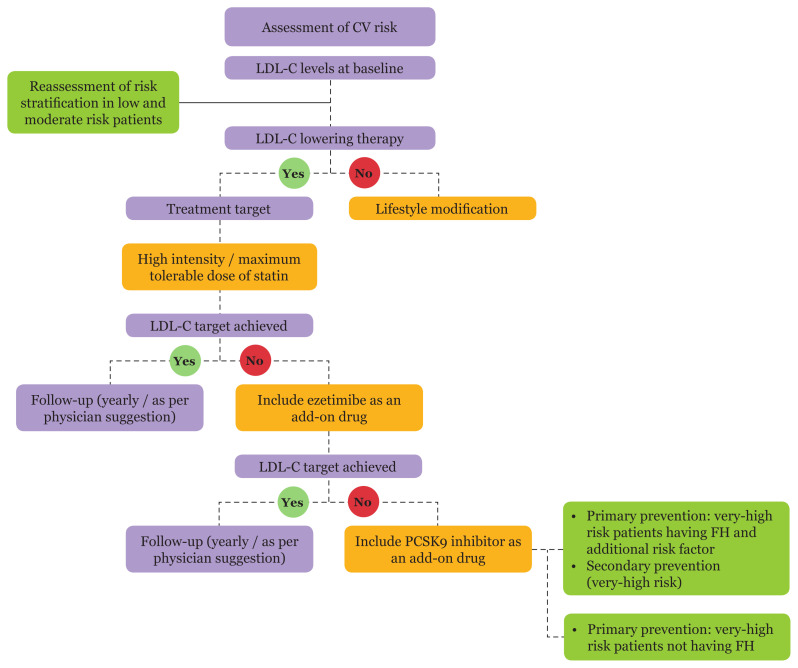
Dyslipidaemia management algorithm for lowering LDL-C levels (24) (Adapted with modifications from 2019 ESC/EAS Guidelines for the Management of Dyslipidaemias). CV = cardiovascular; FH = familial hypercholesterolaemia; LDL-C = low-density lipoprotein cholesterol; PCSK9 = proprotein convertase subtilisin/kexin type 9.

Treatment algorithms help clinicians to select the appropriate treatment modalities for attaining LDL-C goals (Figure 1). Most very high-risk patients fail to achieve LDL-C goals with monotherapy. Hence, combination therapy is recommended for these patients. They are treated with a maximum tolerable dose of statin in combination with ezetimibe or PCSK9 inhibitors as required to lower the LDL-C levels to target (24).

#### Benefits of Lowering LDL-C

Mounting evidence from published literature advocates that lowering LDL-C level leads to overall CV benefits. Aggressive lowering of LDL-C in extreme risk and very high-risk patients results in further reduction in CV events (27, 28).

The landmark clinical trials with lipid-lowering agents that substantiated the benefits of lowering LDL-C levels include Further CV Outcomes Research with PCSK9 Inhibition in Subjects with Elevated Risk (FOURIER), ODYSSEY OUTCOMES and Open Label Study of Long-Term Evaluation against LDL-C (OSLER) (29–31). The FOURIER trial was a randomised, double-blind trial including ASCVD patients (*N* = 27,564) with high LDL-C levels (> 1.8 mmol/L) and receiving statin therapy. The randomised patients were given evolocumab [a monoclonal antibody that blocks proprotein convertase subtilisin–kexin type 9 (PCSK9)] or a placebo. The median baseline LDL-C was 2.4 mmol/L. The least-squares mean percentage reduction in LDL-C levels at week 48 with evolocumab was 59% compared to placebo (*P* < 0.001). Lowering of LDL-C levels to a median of 0.78 mmol/L further reduced the risk of CV events. The risk of the primary composite endpoint of CV death, myocardial infarction (MI), stroke, hospitalisation for unstable angina or coronary revascularisation was significantly reduced by evolocumab (*P* < 0.001). Similarly, there was a significant reduction in the risk of the secondary composite endpoint of CV death, myocardial infarction or stroke by evolocumab (*P* < 0.001) (29). The randomised, double-blind, multicentre ODYSSEY OUTCOMES trial was conducted on patients (*N* = 18,924) with ACS who had a mean baseline LDL-C level of 2.38 ± 0.80 mmol/L and were receiving high dose statin therapy. In intention-to-treat analysis, the mean LDL-C in the alirocumab group was 1.0 mmol/L and 1.7 mmol/L at 4 months and 48 months, respectively. Alirocumab significantly reduced the risk of a composite end-point event (*P* < 0.001). Those treated with alirocumab had a lower risk of major adverse CV events (30).

The OSLER-1 trial on lipid effects and exposure-dependent safety of evolocumab with a follow-up of 5 years (*N* = 1,324) found a decrease in the baseline LDL-C from 3.6 mmol/L to 1.6 mmol/L. About 30% (*n* = 393) and about 7% (*n* = 91) patients achieved LDL-C levels of < 1.0 mmol/L and < 0.6 mmol/L, respectively. The primary composite outcome of death, myocardial infarction, unstable angina, coronary revascularisation, stroke, transient ischaemic attack and chronic heart failure hospitalisation at 1 year was significantly lower in the evolocumab group as compared to the standard of care group (0.95% versus 2.18%; *P* = 0.003) (31).

Several recent randomised clinical trials have reported very low achieved LDL-C levels in patients with dyslipidaemia (Table 2). A meta-analysis revealed 19% relative risk reduction for major vascular events with a 1 mmol/L reduction in LDL-C levels. This risk reduction was independent of baseline LDL-C levels (32). Similar results were seen in a meta-analysis of 11 randomised clinical trials on patients (*N* = 130,070) treated with intensive and less intensive lipid-lowering therapy. The patients were administered statins, ezetimibe or PCSK9 inhibitor and were followed up for 2 years. The group with lower LDL-C (< 1.8 mmol/L) achieved a median LDL-C level of 1.6 mmol/L; whereas the achieved LDL-C level in the group with higher LDL-C (≥ 1.8 mmol/L) was 2.7 mmol/L. The all-cause mortality [risk ratio (RR) = 0.94; absolute risk differences (ARD) = −1.56 incident cases per 1000 person-years (−2.76, −0.35)] and CV mortality [RR = 0.90; ARD = −1.49 incident cases per 1000 person-years (−2.67, −0.31)] were reduced further in the lower LDL-C group (< 1.8 mmol/L) (33). Intensive reduction in LDL-C levels was associated with further reduction in CV events (34).

Maintaining optimal lipid levels from a very early age will reduce the risk of ASCVD. The magnitude and cumulative exposure duration to LDL-C and other lipoproteins are responsible for their deleterious effects. The cumulative LDL-C exposure is estimated by multiplying the age by plasma LDL-C concentration and gives an approximate estimate of the total atherosclerotic plaque burden. Cumulative risk of MI is measured on a log scale. As the LDL-C level maintained throughout life increases and the age at which the cumulative LDL-C exposure threshold exceeds 5,000 mg-years, the risk of ACS happens earlier in life. Once the threshold is exceeded the atherosclerotic plaque burden will increase the risk of MI. Hence, maintaining lower cumulative exposure to LDL-C can prevent/minimise the risk of ASCVD events (35). Another study has proved that the risk of having CVD events in future is associated with cumulative exposure to LDL-C. This was calculated from the area under the LDL-C versus age curve and the risk was determined by the time course of area accumulation (3). A consistent dose-dependent link between the absolute amount of LDL-C exposure and the risk of ASCVD was observed by Ference et al. (14) emphasising the importance of treating dyslipidaemia early, which may lead to the need for less severe treatment in the long run.

#### Safety Profile in Lower LDL-C Scenario

There have been concerns about possible side effects of very low LDL-C levels. These concerns were addressed in the breakthrough clinical trials that evaluated the efficacy and safety outcomes of lowered LDL-C levels by lipid-lowering agents. In the FOURIER trial, there was no significant difference in adverse events between the evolocumab and placebo groups (29). In the ODYSSEY OUTCOMES trial, the occurrence of the adverse events was similar in the alirocumab group and placebo (30). In the OSLER-1 trial, there was no difference in the rate of adverse events (AEs) reported in these patients compared to those with LDL-C ≥ 1.0 mmol/L. Neutralising antibodies were not detected and evolocumab was found to be safe and well-tolerated (31).

The Improved Reduction of Outcomes: Vytorin Efficacy International Trial (IMPROVE-IT) has shown that individuals who achieved very low LDL-C level (< 0.8 mmol/L) had a similar safety profile as patients with high LDL-C levels. The adjusted hazard ratio (HR) of the primary efficacy composite of major coronary events, or stroke was significantly lower in patients with LDL-C level < 0.8 mmol/L (adjusted HR = 0.79; *P* = 0.001) compared to those with ≥ 1.8 mmol/L (34).

A meta-analysis performed on two groups (one group was on statin therapy and another group on non-statin therapy) also underscores the safety of reducing LDL-C levels. The data for statin therapy was extracted from cholesterol treatment trialists’ collaboration (CTTC). Data obtained from IMPROVE-IT, FOURIER and Randomised Evaluation of the Effects of Anacetrapib through Lipid Modification (REVEAL) trials were considered for non-statin therapy. The overall RR was 0.79 per 1 mmol/L reduction in LDL-C for both statins and non-statins (*P* < 0.001). Lowering of baseline LDL-C levels (from 1.6 mmol/L to 0.5 mmol/L) reduced the risk for CV events and was safe with no serious AEs, elevated aminotransferases, new-onset diabetes (NOD), haemorrhagic stroke, cancer or myalgias. Therefore, irrespective of the type of lipid-lowering agents, a reduction in 1 mmol/L LDL-C was primarily responsible for better clinical outcomes (36). Another meta-analysis of 27 randomised trials (*N* = 134,537) revealed that in comparison to control a reduction in 1 mmol/L LDL-C by statin resulted in an absolute reduction in major vascular events (3.27% versus 4.04% per annum) such as stroke (0.67% versus 0.78% per annum), major coronary event (1.45% versus 1.87% per annum) and coronary revascularisation (1.55% versus 1.98% per annum) (37). The lowering of LDL-C as low as 0.78 mmol/L (slope = 0.011; *P* = 0.779) did not increase the risk for haemorrhagic stroke (38).

Published reports demonstrated that lowering of LDL-C with evolocumab was found to be safe and well-tolerated as the frequency of AE was minimum. There was no effect on glucose metabolism and neutralising antibodies were not detected (39, 40). Likewise, another study on alirocumab found no increase in hemorrhagic stroke in patients with ACS and dyslipidaemia, emphasising the safety of lower LDL-C goals (41). These promising data on the safety of very low LDL-C support the hypothesis that ‘lower is better’.

### Rationale for lowering LDL-C for longer duration

- Lowering of LDL-C level results in significant reduction in atherosclerotic CV events (27, 28).
- Intensive lowering of LDL-C levels to achieve treatment goals is well-tolerated and safe without severe adverse events (41).
- The duration of cumulative LDL-C exposure is associated with future CV events. In young individuals, the risk for ASCVD can be reduced by maintaining ideal lipid levels from an early age so that they can benefit from lifelong exposure to reduced LDL-C levels (3, 35).

### Challenges in the Current Management of Dyslipidaemia

Lowering LDL-C to even 0.3 mmol/L was associated with an increase in CV benefits without attaining a plateau stage (27). Patients with very high and extreme risk of ASCVD require aggressive treatment modalities to reduce atherogenic lipoprotein cholesterol to prevent major atherosclerotic CV events. Targeting to achieve lower LDL-C levels with moderate and high-intensity statin monotherapy or combination therapy with add-on non-statins is the generally followed treatment regimen (25).

The cost-effectiveness, CV clinical benefits and co-morbid conditions need to be considered before achieving very low LDL-C levels (27). The safety and tolerability of PCSK9 inhibitors along with LDL-C lowering to exceptional levels and related CV benefits make them the preferred treatment option for dyslipidaemia (42). However, high cost is responsible for the limited use of PSCK9 inhibitors (43).

There are many plausible reasons for not achieving LDL-C goals. Lack of adherence to medication is a contributing factor. Poor adherence deters the benefits of lipid-lowering therapy and the patient becomes vulnerable to CV events. The lack of affordability of medicines, polypharmacy, forgetfulness, doctor-patient relationship, inability to understand label instructions or the need to take the medication are some of the factors responsible for poor adherence. Simple to follow regimens, clear instructions, family support, promoting self-monitoring, providing appointments or follow-up visit reminders and patient education could help improve adherence and compliance. Physicians should follow lipid treatment guidelines and reference values in all laboratories need to be standardised based on the recommended Malaysian CPG (20).

Another reason for not achieving LDL-C targets is the adverse effect of statin therapy. The most common are statin-associated muscle symptoms (SAMS) which result in low drug adherence and high drug discontinuation rate. This can be managed with dose adjustment and patient counselling (44). Analysis of the JUPITER trial identified statin therapy as a risk for NOD. Some patients are intolerant to statins and develop skeletal muscle-related symptoms leading to treatment discontinuation. There is an increased risk of development of diabetes associated with statin use; however, the benefits of therapy outweigh the risk for diabetes (45).

The WHO had initiated a global strategy program to strengthen the surveillance of NCD in member countries including Malaysia (46). Presently, a dynamic monitoring system for NCD surveillance is actively present in Malaysia for indicators that are tracked and monitored by the Global Monitoring Framework. The list of 25 indicators includes ‘raised total cholesterol’ under the monitoring of risk/exposure factors. Even though the NCD surveillance system in Malaysia is quite proficient, there is still a deficiency of necessary data for monitoring the framework’s health system metrics. There is a lack of an interconnected electronic medical record (EMR) system between public and private healthcare facilities. Despite the availability of web-based systems, data collection largely depends on manual systems (47).

Enhancing public information and mass communication systems could promote greater awareness among the population. Women in Malaysia seem to have good knowledge and attitude towards CVD risk factors (48). Of note, compared to international data, there is gender bias with respect to care in terms of health care providers not being as attuned to CVD in females compared to males.

### Newer therapies on the horizon

Nowadays other than statins, PCSK9 inhibitors, adenosine triphosphate-citrate lyase inhibitors and oligonucleotides are used as lipid-lowering agents. The PCSK9, a serine protease synthesised by liver is responsible for elevated LDL-C levels (49). PCSK9 inhibitors are human monoclonal antibodies directed against PCSK9 enzyme resulting in the up-regulation of LDL-C receptors on the cell surface. This leads to increased LDL-C uptake into cells thereby lowering the serum LDL-C levels and CV risks (50). The US Food and Drug Administration (FDA) has approved two PCSK9 inhibitors, alirocumab and evolocumab. Alirocumab (Praluent) was approved for adult patients with heterozygous familial hypercholesterolemia (HeFH) or established ASCVD requiring additional lowering of LDL-C. Evolocumab (Repatha) is to be used in adult patients with HeFH, homozygous familial hypercholesterolaemia or clinical ASCVD requiring further lowering of LDL-C (51). Unfortunately for HeFH, control rates in Malaysia were lower in comparison to other countries (52).

Apart from PCSK9 inhibitors, small interfering RNA (siRNA) molecule can be used to curtail the hepatic production of PCSK9. Inclisiran is one such long-acting, synthetic siRNA molecule that binds to RNA-induced silencing complex (RISC), thereby inhibiting the translation of PCSK9 messenger RNA (mRNA) (49). Inclisiran injection has been approved recently by US FDA for adults with HeFH or clinical ASCVD along with diet and maximally tolerated statin therapy (53). Moreover, the European Medicines Agency (EMA) had also approved inclisiran in 2020 for adults with primary hypercholesterolaemia or mixed dyslipidaemia along with a low-fat diet. Inclisiran can be used in patients on maximally tolerated statins and those who are not able to take the statins (54). Inclisiran is administered as a subcutaneous injection initially, followed by same dose at 3 months and then once every 6 months (53).

Another LDL-C lowering agent is bempedoic acid which is a prodrug. It inhibits the enzyme adenosine triphosphate-citrate lyase (ACL) in the liver and reduces the availability of precursors for fatty acid and cholesterol synthesis (55). Bempedoic acid (ETC-1002) also activates 5′-adenosine monophosphate–activated protein kinase, thereby effectively reducing serum LDL-C levels (56, 57). Bempedoic acid was approved by the US FDA for adults with HeFH or established ASCVD who require additional lowering of LDL-C to achieve treatment goals (55). There is an increasing interest in the use of omega-3 fatty acids supplements. Further research is needed to evaluate the effect of these supplements on reducing LDL-C levels (57).

### Guideline Updates

#### Recent Advances in International Recommendations

The 2018 ACC/AHA Guideline on the Management of Blood Cholesterol recommended a ≥ 50% reduction in LDL-C from baseline (25). Furthermore, the 2019 ESC/EAS Guideline on the Management of Dyslipidaemias recommended more intensive LDL-C reduction. For high-risk patients, ≥ 50% reduction from baseline and < 1.8 mmol/L LDL-C goal was recommended. For very high-risk patients on primary and secondary prevention ≥ 50% reduction from baseline and < 1.4 mmol/L LDL-C level was recommended. In the subset of very high-risk patients, an LDL-C goal of < 1.0 mmol/L was recommended for ASCVD patients on maximally tolerated statin therapy who had second CV events in a duration of 2 years (Table 1) (24). These guidelines recognise all ASCVD patients to be at very high risk for developing adverse CV events in the future. In comparison to 2018 ACC/AHA guidelines, an aggressive LDL-C goal is recommended for these patients (58).

Based on the consensus statement by the American Association of Clinical Endocrinologists and American College of Endocrinology on the management of dyslipidaemia and prevention of CVD algorithm, the target LDL-C for extreme risk, very high-risk and high-risk groups were < 1.4 mmol/L, < 1.8 mmol/L and < 2.6 mmol/L, respectively (59). The 2019 ESC Guidelines on diabetes, pre-diabetes and CVD in patients with type 2 diabetes mellitus (T2DM) have recommended LDL-C goal of < 1.4 mmol/L and ≥ 50% LDL-C reduction in T2DM patients with very high CV risk. LDL-C reduction of ≥ 50% and target LDL-C of < 1.8 mmol/L to be advocated for high CV risk patients with T2DM (60).

According to the 2019 ACC/AHA Guideline on the Primary Prevention of CV Disease, ≥ 50% LDL-C reduction was proposed for high-risk patients with ≥ 20% 10-year ASCVD risk. In intermediate-risk patients with a 10-year ASCVD risk of 7.5% to < 20%, the recommended reduction was ≥ 30% (61).

A position paper endorsed by the International Lipid Expert Panel recommended maximally tolerated intensive statin therapy for statin-treated patients with LDL-C < 2.5 mmol/L and high dose statins for statin-naive patients with LDL-C < 3.0 mmol/L. The treatment goal was a 50% reduction of LDL-C or target LDL-C < 1.4 mmol/L. For statin treated patients (LDL-C 2.5 mmol/L–7.5 mmol/L) or statin naive patients (LDL-C 3.0 mmol/L–7.5 mmol/L), maximally tolerated statin therapy in combination with ezetimibe was recommended to achieve a 50%–80% reduction in LDL-C (target < 1.4 mmol/L). For LDL-C levels > 7.5 mmol/L on admission, LDL-C reduction of > 80% was recommended to attain a target LDL-C of < 1.4 mmol/L. Hence a triple therapy (statin + ezetimibe + PCSK9 inhibitor) might be required to achieve the goal (62).

#### Recent Advances in Local Guidelines

There has been a recent update of two clinical guidelines in Malaysia wherein the target LDL-C goals for the different risk categories have been revised. The 2020 Malaysian CPG on the management of T2DM recommended a target of LDL-C < 1.4 mmol/L in very high-risk patients (with diabetes and established CVD or target organ damage or ≥ 3 risk factors). However, the recommended LDL-C goals for high-risk patients (with ≥ 10 years of diabetes without target organ damage and no other risk factors) and for moderate-risk patients (< 50 years old with < 10 years of T2DM without additional risk factors) were < 1.8 mmol/L and < 2.6 mmol/L, respectively (63). The target LDL-C recommended by the 2020 Malaysian CPG on the management of T2DM is similar to that recommended by the 2019 ESC/EAS Guideline on the management of dyslipidaemias.

Furthermore, the 2020 Malaysian CPG on the management of ischaemic stroke recommended LDL-C target of < 1.8 mmol/L for high-risk group and in patients with prior ischaemic stroke (secondary prevention of stroke). Lowering of LDL-C to < 3.4mmol/L was recommended for intermediate and low-risk populations (64).

### Future Directions in Management of Dyslipidaemia

Non-communicable diseases have become the leading cause of morbidity and mortality. Surveillance by NCD could be improved through implementing electronic records in healthcare facilities in Malaysia. Reducing the burden of NCDs will improve the economic status and ensure healthy living for people worldwide. Ensuring adherence to CPG and addressing the knowledge gaps through training sessions for primary care physicians are recommended for better management of dyslipidaemia (65). This highlights the need for programmes and workshops to spread awareness about dyslipidaemia and its management amongst the local population in Malaysia (66). Asian countries need to evaluate, accept, and implement the essential recommendations from ESC/EAS and ACC/AHA guidelines for dyslipidaemia management in their respective populations. These countries need to update and tailor their CPG in a way suitable for the target population (67).

Considering the advent of several new clinical data, gaps in the knowledge as well as non-adherence to guidelines make it necessary to update the guidelines regularly to re-evaluate the LDL-C targets as well as consider treatment modalities beyond statins to achieve these new targets. This would facilitate the dissemination of awareness about the management of dyslipidaemia and related risks.

## Figures and Tables

**Table 1:** Risk stratification and recommendations of LDL-C goals

Clinical practice guidelines	Risk stratification	Risk categories	LDL-C goals (mmol/L, % reduction from baseline)
2017 Malaysian Clinical Practice Guidelines on the Management of Dyslipidaemia (20)	Very high CV risk	Established CVD; diabetes with proteinuria/one major risk factor (smoking/hypertension/dyslipidaemia); ≥ Stage 4 CKD not on dialysis (GFR < 30 mL/min/1.73m^2^)	< 1.8 or > 50%
	High CV risk	Diabetes (no target organ damage); elevated levels of risk factors (LDL-C > 4.9 mmol/L, BP > 180/110 mmHg); Stage 3 CKD (GFR ≥ 30–< 60 mL/min/1.73m^2^); 10-year CVD risk of > 20% (FRS-CVD score)	≤ 2.6 or > 50%
	Intermediate CV risk	10-year CVD risk of 10%–20% (FRS-CVD score)	< 3.0
	Low CV risk	10-year CVD risk of < 10% (FRS-CVD score)	< 3.0

2018 AHA/ACC Guideline on the Management of Blood Cholesterol (25)	Very high risk ASCVD	History of multiple major ASCVD events or one major ASCVD event and multiple high-risk conditions	≥ 50%
	ASCVD not very high risk	Age ≥ 65 years old; HeFH; history of prior CABG/PCI; DM; Hypertension; CKD (eGFR 15–59 mL/min/1.73 m2); currently smoking; LDL-C ≥ 2.6 mmol/L despite maximally tolerated statin and ezetimibe; history of congestive HF	≥ 50%
	High risk ASCVD on primary prevention	LDL-C ≥ 4.9 mmol/L (hypercholesterolemia); diabetes; 40 years old–75 years old of age	≥ 50%
	Intermediate risk	40 years old–75 years of age without DM; 10-year ASCVD risk of 7.5%–19.9%	30%–49%

2019 ESC/EAS Guideline on the Management of Dyslipidaemias (24)	Very high risk ASCVD on maximally tolerated statin therapy with second CV event in 2 years		< 1.0
	Very high risk patients	Documented ASCVD; DM with target organ damage/three major risk factors/T1DM duration > 20 years; FH with ASCVD/additional major risk factor; severe CKD (eGFR < 30 mL/min/1.73 m^2^); 10-year fatal CVD risk of ≥ 10% (calculated SCORE)	< 1.4 and ≥ 50%
	High risk	DM (no target organ damage), DM duration ≥ 10 years/additional risk factor; high levels of single risk factors (LDL-C > 4.9 mmol/L, TC > 8 mmol/L, BP ≥180/110 mmHg); FH without additional major risk factors; moderate CKD (eGFR 30–59 mL/min/1.73 m^2^); 10-year fatal CVD risk of ≥ 5% and < 10% (calculated SCORE)	< 1.8 and ≥ 50%
	Moderate risk	T1DM (< 35 years old of age) or T2DM (< 50 years old of age), DM duration < 10 years, no additional risk factors; 10-year fatal CVD risk of ≥ 1 % and < 5% (calculated SCORE)	< 2.6
	Low risk	10-year fatal CVD risk of < 1% (calculated SCORE)	< 3.0

Abbreviations: CV = cardiovascular; ACC/AHA = American College of Cardiology/American Heart Association; ESC/EAS = European Society of Cardiology/European Atherosclerosis Society; ASCVD = atherosclerotic cardiovascular disease; CVD = cardiovascular disease; CKD = chronic kidney disease; GFR = glomerular patient filtration rate; eGFR = estimated glomerular patient filtration rate; FRS = Framingham Risk Score; HeFH = heterozygous familial hypercholesterolemia; CABG = coronary artery bypass surgery; PCI = percutaneous coronary intervention; HF = heart failure; LDL-C = low-density lipoprotein cholesterol; DM = diabetes mellitus; T1DM = type 1 diabetes mellitus; T2DM = type 2 diabetes mellitus; BP = blood pressure; FH = familial hypercholesterolaemia; TC = total cholesterol; SCORE = Systematic Coronary Risk Estimation

**Table 2:** Clinical trials on very low achieved LDL-C levels

Trials	Lipid lowering agents	Follow up duration (years)	Lowest achieved LDL-C levels (mmol/L)
OSLER-1 trial (31)	SOC/evolocumab	5	< 0.6
Insights from FOURIER trial (68)	statin/evolocumab	2.2 (median)	0.3
Secondary ad hoc analysis of FOURIER trial (69)	statin/evolocumab	2.2 (median)	0.5
Prespecified analysis of FOURIER trial (17)	statin/evolocumab	2.2 (median)	0.8
RCT (70)	statin/alirocumab, evolocumab	1	< 0.8
Prespecified analysis of ODYSSEY outcomes trial (41)	statin/alirocumab	2.8 (median)	< 0.6
ODYSSEY outcomes trial (30)	statin/alirocumab	2.8 (median)	1.0

Abbreviations: SOC = standard of care; LDL-C = low-density lipoprotein cholesterol; OSLER-1 = open label study of long-term evaluation against LDL-C; FOURIER = further cardiovascular outcomes research with PCSK9 inhibition in subjects with elevated risk; RCT = randomised controlled trial; ODYSSEY = evaluation of cardiovascular outcomes after an acute coronary syndrome during treatment with alirocumab

## References

[1] World Health Organization (WHO) (2021). Cardiovascular diseases (CVDs) [Internet].

[2] Roth GA, Johnson C, Abajobir A, Abd-Allah F, Abera SF, Abyu G (2017). Global, regional, and national burden of cardiovascular diseases for 10 causes, 1990 to 2015. J Am Coll Cardiol.

[3] Domanski MJ, Tian X, Wu CO, Reis JP, Dey AK, Gu Y (2020). Time course of LDL cholesterol exposure and cardiovascular disease event risk. J Am Coll Cardiol.

[4] Benjamin EJ, Virani SS, Callaway CW, Chamberlain AM, Chang AR, Cheng S (2018). Heart disease and stroke statistics-2018 update: a report from the American heart association. Circulation.

[5] Mohamed-Yassin MS, Baharudin N, Daher AM, Abu Bakar N, Ramli AS, Abdul-Razak S (2021). High prevalence of dyslipidaemia subtypes and their associated personal and clinical attributes in Malaysian adults: the REDISCOVER study. BMC Cardiovasc Disord.

[6] Hajar R (2017). Risk factors for coronary artery disease: historical perspectives. Heart Views.

[7] World Health Organization (WHO) (2018). Noncommunicable diseases country profiles [Internet].

[8] Malaysia Department of Statistics (2020). Statistics on causes of death, Malaysia, 2020 [Internet].

[9] National Institute of Health (NIH) (2019). National Health and Morbidity Survey 2019 noncommunicable diseases, healthcare demand, and health literacy: key findings [Internet].

[10] Malaysia NHAo (2016). Annual Report of the NCVD-PCI Registry 2015–2016 [Internet].

[11] Malaysia NHAo (2015). Summary—Annual report NCVD-ACS registry 2006–2015 [Internet].

[12] Goldstein JL, Brown MS (2015). A century of cholesterol and coronaries: from plaques to genes to statins. Cell.

[13] Sandesara PB, Virani SS, Fazio S, Shapiro MD (2019). The forgotten lipids: triglycerides, remnant cholesterol, and atherosclerotic cardiovascular disease risk. Endocr Rev.

[14] Ference BA, Ginsberg HN, Graham I, Ray KK, Packard CJ, Bruckert E (2017). Low-density lipoproteins cause atherosclerotic cardiovascular disease. 1. Evidence from genetic, epidemiologic, and clinical studies. A consensus statement from the European Atherosclerosis Society Consensus Panel. Eur Heart J.

[15] Ray KK, Del Prato S, Müller-Wieland D, Cariou B, Colhoun HM, Tinahones FJ (2019). Alirocumab therapy in individuals with type 2 diabetes mellitus and atherosclerotic cardiovascular disease: analysis of the ODYSSEY DM-DYSLIPIDEMIA and DM-INSULIN studies. Cardiovasc Diabetol.

[16] Murphy SA, Pedersen TR, Gaciong ZA, Ceska R, Ezhov MV, Connolly DL (2019). Effect of the PCSK9 inhibitor evolocumab on total cardiovascular events in patients with cardiovascular disease: a prespecified analysis from the FOURIER trial. JAMA Cardiol.

[17] Sabatine MS, Leiter LA, Wiviott SD, Giugliano RP, Deedwania P, De Ferrari GM (2017). Cardiovascular safety and efficacy of the PCSK9 inhibitor evolocumab in patients with and without diabetes and the effect of evolocumab on glycaemia and risk of new-onset diabetes: a prespecified analysis of the FOURIER randomised controlled trial. Lancet Diabetes Endocrinol.

[18] Pownall HJ, Gotto AM (2019). Cholesterol: can’t live with it, can’t live without it. Methodist Debakey Cardiovasc J.

[19] World Health Organization (WHO) (2007). Prevention of cardiovascular disease guidelines for assessment and management of cardiovascular risk [Internet].

[20] Ministry of Health (MoH) Malaysia (2017). 5th edition of the CPG on the management of dyslipidaemia [Internet].

[21] Garg N, Muduli SK, Kapoor A, Tewari S, Kumar S, Khanna R (2017). Comparison of different cardiovascular risk score calculators for cardiovascular risk prediction and guideline recommended statin uses. Indian Heart J.

[22] Selvarajah S, Kaur G, Haniff J, Cheong KC, Hiong TG, van der Graaf Y (2014). Comparison of the Framingham risk score, SCORE and WHO/ISH cardiovascular risk prediction models in an Asian population. Int J Cardiol.

[23] Chia YC, Gray SY, Ching SM, Lim HM, Chinna K (2015). Validation of the Framingham general cardiovascular risk score in a multiethnic Asian population: a retrospective cohort study. BMJ Open.

[24] Mach F, Baigent C, Catapano AL, Koskinas KC, Casula M, Badimon L (2020). 2019 ESC/EAS Guidelines for the management of dyslipidaemias: lipid modification to reduce cardiovascular risk. Eur Heart J.

[25] Grundy SM, Stone NJ, Bailey AL, Beam C, Birtcher KK, Blumenthal RS (2019). 2018 AHA/ACC/AACVPR/AAPA/ABC/ACPM/ADA/AGS/APhA/ASPC/NLA/PCNA Guideline on the management of blood cholesterol: a report of the American College of Cardiology/American Heart Association task force on clinical practice guidelines. Circulation.

[26] Ministry of Health (MoH) Malaysia (2017). Clinical practice guidelines on primary & secondary prevention of cardiovascular disease 2017 [Internet].

[27] Karagiannis AD, Mehta A, Dhindsa DS, Virani SS, Orringer CE, Blumenthal RS (2021). How low is safe? The frontier of very low (<30 mg/dL) LDL cholesterol. Eur Heart J.

[28] Rosenblit PD (2019). Lowering targeted atherogenic lipoprotein cholesterol goals for patients at ‘extreme’ ASCVD risk. Curr Diab Rep.

[29] Sabatine MS, Giugliano RP, Keech AC, Honarpour N, Wiviott SD, Murphy SA (2017). Evolocumab and clinical outcomes in patients with cardiovascular disease. N Engl J Med.

[30] Schwartz GG, Steg PG, Szarek M, Bhatt DL, Bittner VA, Diaz R (2018). Alirocumab and cardiovascular outcomes after acute coronary syndrome. N Engl J Med.

[31] Koren MJ, Sabatine MS, Giugliano RP, Langslet G, Wiviott SD, Ruzza A (2019). Long-term efficacy and safety of evolocumab in patients with hypercholesterolemia. J Am Coll Cardiol.

[32] Wang N, Fulcher J, Abeysuriya N, Park L, Kumar S, Di Tanna GL (2020). Intensive LDL cholesterol-lowering treatment beyond current recommendations for the prevention of major vascular events: a systematic review and meta-analysis of randomised trials including 327,037 participants. Lancet Diabetes Endocrinol.

[33] Khan SU, Khan MU, Virani SS, Khan MS, Khan MZ, Rashid M (2020). Efficacy and safety for the achievement of guideline-recommended lower low-density lipoprotein cholesterol levels: a systematic review and meta-analysis. Eur J Prev Cardiol.

[34] Giugliano RP, Wiviott SD, Blazing MA, De Ferrari GM, Park JG, Murphy SA (2017). Long-term safety and efficacy of achieving very low levels of low-density lipoprotein cholesterol: a prespecified analysis of the IMPROVE-IT trial. JAMA Cardiol.

[35] Ference BA, Graham I, Tokgozoglu L, Catapano AL (2018). Impact of lipids on cardiovascular health: JACC health promotion series. J Am Coll Cardiol.

[36] Sabatine MS, Wiviott SD, Im K, Murphy SA, Giugliano RP (2018). Efficacy and safety of further lowering of low-density lipoprotein cholesterol in patients starting with very low levels: a meta-analysis. JAMA Cardiol.

[37] Mihaylova B, Emberson J, Blackwell L, Keech A, Simes J, Barnes EH (2012). The effects of lowering LDL cholesterol with statin therapy in people at low risk of vascular disease: meta-analysis of individual data from 27 randomised trials. Lancet.

[38] Shin J, Chung JW, Jang HS, Lee J, Hong KS, Bang OY (2019). Achieved low-density lipoprotein cholesterol level and stroke risk: a meta-analysis of 23 randomised trials. Eur J Prev Cardiol.

[39] Koren MJ, Sabatine MS, Giugliano RP, Langslet G, Wiviott SD, Kassahun H (2017). Long-term low-density lipoprotein cholesterol-lowering efficacy, persistence, and safety of evolocumab in treatment of hypercholesterolemia: results up to 4 years from the open-label OSLER-1 extension study. JAMA Cardiol.

[40] Rosenson RS, Daviglus ML, Handelsman Y, Pozzilli P, Bays H, Monsalvo ML (2019). Efficacy and safety of evolocumab in individuals with type 2 diabetes mellitus: primary results of the randomised controlled BANTING study. Diabetologia.

[41] Jukema JW, Zijlstra LE, Bhatt DL, Bittner VA, Diaz R, Drexel H (2019). Effect of alirocumab on stroke in ODYSSEY OUTCOMES. Circulation.

[42] Iqbal Z, Dhage S, Mohamad JB, Abdel-Razik A, Donn R, Malik R (2019). Efficacy and safety of PCSK9 monoclonal antibodies. Expert Opin Drug Saf.

[43] Tomlinson B, Chan P, Zhang Y, Lam CWK (2020). Efficacy and safety of add on therapies in patients with hypercholesterolemia undergoing statin therapy. Expert Opin Pharmacother.

[44] Laufs U, Scharnagl H, Halle M, Windler E, Endres M, März W (2015). Treatment options for statin-associated muscle symptoms. Dtsch Arztebl Int.

[45] Ridker PM, Pradhan A, MacFadyen JG, Libby P, Glynn RJ (2012). Cardiovascular benefits and diabetes risks of statin therapy in primary prevention: an analysis from the JUPITER trial. Lancet.

[46] World Health Organization (WHO) (2010). Global status report on noncommunicable diseases 2010 [Internet].

[47] Chandran A, Selva Kumar S, Hairi NN, Low WY, Mustapha FI (2021). Non-communicable disease surveillance in Malaysia: an overview of existing systems and priorities going forward. Front Public Health.

[48] Mohammad NB, Rahman NAA, Haque M (2018). Knowledge, attitude, and practice regarding the risk of cardiovascular diseases in patients attending outpatient clinic in Kuantan, Malaysia. J Pharm Bioallied Sci.

[49] Kosmas CE, Muñoz Estrella A, Sourlas A, Silverio D, Hilario E, Montan PD (2018). Inclisiran: a new promising agent in the management of hypercholesterolemia. Diseases.

[50] Kosmas CE, Skavdis A, Sourlas A, Papakonstantinou EJ, Peña Genao E, Echavarria Uceta R (2020). Safety and tolerability of PCSK9 inhibitors: current insights. Clin Pharmacol.

[51] Chaudhary R, Garg J, Shah N, Sumner A (2017). PCSK9 inhibitors: a new era of lipid lowering therapy. World J Cardiol.

[52] Pang J, Chan DC, Hu M, Muir LA, Kwok S, Charng MJ (2019). Comparative aspects of the care of familial hypercholesterolemia in the ‘Ten Countries Study’. J Clin Lipidol.

[53] Food and Drug Administration (FDA) (2021). FDA approves add-on therapy to lower cholesterol among certain high-risk adults [Internet].

[54] EMA (2020). Leqvio (inclisiran): an overview of Leqvio and why it is authorised in the EU [Internet].

[55] Marrs JC, Anderson SL (2020). Bempedoic acid for the treatment of dyslipidemia. Drugs Context.

[56] Lemus HN, Mendivil CO (2015). Adenosine triphosphate citrate lyase: emerging target in the treatment of dyslipidemia. J Clin Lipidol.

[57] Jang AY, Lim S, Jo SH, Han SH, Koh KK (2021). New trends in dyslipidemia treatment. Circ J.

[58] Raygor V, Khera A (2020). New recommendations and revised concepts in recent guidelines on the management of dyslipidemias to prevent cardiovascular disease: the 2018 ACC/AHA and 2019 ESC/EAS guidelines. Curr Cardiol Rep.

[59] Handelsman Y, Jellinger PS, Guerin CK, Bloomgarden ZT, Brinton EA, Budoff MJ (2020). Consensus statement by the American Association of Clinical Endocrinologists and American College of Endocrinology on the management of dyslipidemia and prevention of cardiovascular disease algorithm - 2020 executive summary. Endocr Pract.

[60] Cosentino F, Grant PJ, Aboyans V, Bailey CJ, Ceriello A, Delgado V (2020). 2019 ESC guidelines on diabetes, pre-diabetes, and cardiovascular diseases developed in collaboration with the EASD. Eur Heart J.

[61] Arnett DK, Blumenthal RS, Albert MA, Buroker AB, Goldberger ZD, Hahn EJ (2019). 2019 ACC/AHA Guideline on the primary prevention of cardiovascular disease: a report of the American College of Cardiology/American Heart Association Task Force on Clinical Practice Guidelines. Circulation.

[62] Banach M, Penson PE, Vrablik M, Bunc M, Dyrbus K, Fedacko J (2021). Optimal use of lipid-lowering therapy after acute coronary syndromes: a position paper endorsed by the international lipid expert panel (ILEP). Pharmacol Res.

[63] Ministry of Health (MoH) (2020). Malaysia clinical practice guidelines on management of type 2 diabetes mellitus.

[64] Ministry of Health (MoH) Malaysia (2020). Clinical practice guidelines on management of ischemic stroke.

[65] Said AH, Chia YC (2017). Awareness, knowledge and practice of dyslipidaemia management among postgraduate primary care trainees in Malaysia: a cross-sectional study. BMJ Open.

[66] Ahmed AAA, Al-Shami AM, Jamshed S, Zawiah M, Elnaem MH, Mohamed Ibrahim MI (2020). Awareness of the risk factors for heart attack among the general public in Pahang, Malaysia: a cross-sectional study. Risk Manag Healthc Policy.

[67] Alshamiri M, Ghanaim MMA, Barter P, Chang KC, Li JJ, Matawaran BJ (2018). Expert opinion on the applicability of dyslipidemia guidelines in Asia and the Middle East. Int J Gen Med.

[68] Bonaca MP, Nault P, Giugliano RP, Keech AC, Pineda AL, Kanevsky E (2018). Low-density lipoprotein cholesterol lowering with evolocumab and outcomes in patients with peripheral artery disease: insights from the FOURIER trial (Further cardiovascular outcomes research with PCSK9 inhibition in subjects with elevated risk). Circulation.

[69] Giugliano RP, Keech A, Murphy SA, Huber K, Tokgozoglu SL, Lewis BS (2017). Clinical efficacy and safety of evolocumab in high-risk patients receiving a statin: secondary analysis of patients with low LDL cholesterol levels and in those already receiving a maximal-potency statin in a randomized clinical trial. JAMA Cardiol.

[70] Rallidis LS, Skoumas I, Liberopoulos EN, Vlachopoulos C, Kiouri E, Koutagiar I (2020). PCSK9 inhibitors in clinical practice: novel directions and new experiences. Hellenic J Cardiol.

